# Feasting on terrestrial organic matter: Dining in a dark lake changes microbial decomposition

**DOI:** 10.1111/gcb.14391

**Published:** 2018-08-26

**Authors:** Amelia Fitch, Chloe Orland, David Willer, Erik J. S. Emilson, Andrew J. Tanentzap

**Affiliations:** ^1^ Department of Plant Sciences University of Cambridge Cambridge UK; ^2^ Natural Resources Canada, Great Lakes Forestry Centre Sault Ste. Marie Ontario

**Keywords:** bacterial production, boreal, carbon cycling, enzyme activity, lake sediments, photo‐oxidation

## Abstract

Boreal lakes are major components of the global carbon cycle, partly because of sediment‐bound heterotrophic microorganisms that decompose within‐lake and terrestrially derived organic matter (t‐OM). The ability for sediment bacteria to break down and alter t‐OM may depend on environmental characteristics and community composition. However, the connection between these two potential drivers of decomposition is poorly understood. We tested how bacterial activity changed along experimental gradients in the quality and quantity of t‐OM inputs into littoral sediments of two small boreal lakes, a dark and a clear lake, and measured the abundance of operational taxonomic units and functional genes to identify mechanisms underlying bacterial responses. We found that bacterial production (BP) decreased across lakes with aromatic dissolved organic matter (DOM) in sediment pore water, but the process underlying this pattern differed between lakes. Bacteria in the dark lake invested in the energetically costly production of extracellular enzymes as aromatic DOM increased in availability in the sediments. By contrast, bacteria in the clear lake may have lacked the nutrients and/or genetic potential to degrade aromatic DOM and instead mineralized photo‐degraded OM into CO_2_. The two lakes differed in community composition, with concentrations of dissolved organic carbon and pH differentiating microbial assemblages. Furthermore, functional genes relating to t‐OM degradation were relatively higher in the dark lake. Our results suggest that future changes in t‐OM inputs to lake sediments will have different effects on carbon cycling depending on the potential for photo‐degradation of OM and composition of resident bacterial communities.

## 1 INTRODUCTION

Inputs of terrestrially derived organic matter (t‐OM) are a major driver in lake ecosystems, but the ecological consequences of changes in their quantity and quality remain poorly understood (Solomon et al., 2015). Nearly a third of all the terrestrial carbon (C) input into inland waters is buried into lake sediments (Tranvik et al., 2009). Much of this burial, especially of larger particulate material, occurs in nearshore environments of small lakes through mechanisms including sedimentation and flocculation, where heavier matter and coagulated dissolved organic matter (DOM) fall out of suspension and accumulate (von Wachenfeldt & Tranvik, 2008; Wurtsbaugh et al., 2002). However, littoral sediments remain understudied relative to pelagic environments, especially in the context of future changes to t‐OM exports. Terrestrial organic matter is increasingly exported into receiving waters, due to mechanisms including declines in anthropogenic sulfur deposition (Evans, Monteith, & Cooper, 2005; Monteith et al., 2007) and increases in terrestrial primary production (Freeman, Ostle, Fenner, & Kang, 2004) and soil decomposition (Findlay, 2005), and should thereby enhance sedimentation (Kritzberg et al., 2014; von Wachenfeldt, Sobek, Bastviken, & Tranvik, 2008). Additionally, northward shifts in deciduous forests (Boisvert‐Marsh, Perie, & Blois, 2014), and the spread of fires and insect outbreaks (Schindler & Lee, 2010), are expected to change the composition of t‐OM that will be buried into receiving waters (e.g., Jaffé et al., 2012).

Although heterotrophic decomposition, particularly by bacteria, is the primary mechanism by which t‐OM is assimilated into aquatic ecosystems (Kirchman, 2013; Solomon et al., 2015), relatively little is known about how and why bacterial activity changes along gradients in the quantity and quality of buried t‐OM. For example, bacterial production (BP) has been found to vary by 70% across freshwater sediments with different DOM concentrations (Cole, Findlay, & Pace, 1988). One explanation for this is that bacteria assimilate low molecular weight (LMW) labile DOM quickly and easily so their production can be elevated in the presence of LMW compounds (Breggren et al., 2010; Kirchman, 2013; Smith & Prairie, 2004). By contrast, high molecular weight (HMW) compounds, including lignin, humic acid, and aromatic molecules, require the production of enzymes that hydrolyze or oxidize complex structures into LMW compounds before they can be assimilated (Burns et al., 2013; Fuchs, Mattias, & Heider, 2011; Kirchman, 2013; Sinsabaugh, Findlay, Franchini, & Fischer, 1997). Therefore, while HMW compounds are thought to be less amenable to microbial processing, the production of extracellular enzymes can allow bacteria to utilize these substrates and access both C and nutrients associated with terrestrially derived OM (Guillemette, McCallister, & Giorgio, 2016; Judd, Crump, & Kling, 2006; Kritzberg et al., 2014; Tranvik, 1988). Extracellular enzyme activity (EEA) should increase with concentrations of t‐OM in lakes with limited LMW exudates (DeAngelis, Allgaier, & Chavarria, 2011; Sinsabaugh, 2010), providing a source of C and nutrients, but may result in less bacterial growth as it comes with a high metabolic cost (Chróst & Siuda, 2002; Sieczko, Maschek, & Peduzzi, 2015). Because extracellular enzymes are energetically costly to produce, bacterial growth may even be stagnant or negative with increasing concentrations of HMW OM (Chróst, 1991).

Environmental conditions, such as sediment light exposure and nutrient availability, will also interact with t‐OM supply to dictate how bacterial communities will respond to future increases in the sedimentation of t‐OM. For example, high light levels at the sediment–water interface can increase the availability of LMW phytoplankton exudates and photo‐oxidize up to 70%–95% of the HMW t‐OM found in lakes into LMW compounds (Hunting et al., 2013; Kirchman, 2013; Ward & Cory, 2016). This process can therefore negate the reliance of bacterial communities on EEAs. By contrast, lakes with lower sediment light exposure will have reduced oxidation of dissolved compounds at the sediment–water interface, increasing their availability to bacteria and their ability to settle into sediments by forming organic particles (von Wachenfeldt et al., 2008). Bacteria may consequently rely on EEAs to acquire LMW C from HMW t‐OM (Kirchman, 2013). Because EEA is the rate‐limiting step for making HMW C available to bacteria, the total pool of available LMW C could also be smaller in these cases (Azam & Cho, 1987; Jørgensen, 1987; Münster, 1985). Enhanced BP from LMW compounds will also depend on an adequate supply of nutrients for building proteins and cells (Goldman, Caron, & Dennett, 1987; Reche, Pace, & Cole, 1998; Russell, 2007; Smith & Prairie, 2004; Sterner, Clasen, Lampert, & Weisse, 1998).

Changes in bacterial functions involved in heterotrophic decomposition, including EEA, may also arise because the ability to degrade HMW compounds is taxonomically restricted (Bertilsson, Eiler, Nordqvist, & Jorgensen, 2007; DeAngelis et al., 2011; Ruiz‐Gonzalez, Nino‐Garcia, Lapierre, & Giorgio, 2015; Zifcakova, Vetrovsky, Howe, & Baldrian, 2016). Consequently, shifts in bacterial community composition with increasing t‐OM inputs will alter bacterial function (Logue et al., 2016). The functioning of whole communities can also change independently of composition if there is a high degree of redundancy among taxonomically distinct lineages (Ruiz‐Gonzalez et al., 2015). However, irrespective of the underlying cause of change, no study has yet connected actual and potential functioning of sediment bacterial communities to the quantity and quality of t‐OM input into lake sediments. Understanding this connection can help improve predictions of future changes in diverse ecosystem processes such as C cycling, food web production, and water quality (Solomon et al., 2015).

Here, our aim was to characterize how bacterial activity changed along fine‐scale gradients in the quality and quantity of t‐OM in the littoral sediments of small boreal lakes. We addressed this aim by analyzing OM from pore water collected at the interface of the sediment and overlying lake water, where t‐OM is primarily deposited (Sobek et al., 2009). We focused on the effect of terrestrially sourced particulate OM as this is the primary way in which t‐OM enters the littoral zone and benthic food web (Scharnweber et al., 2014; Wetzel, 1992). Boreal lakes also have varying water clarity and dissolved organic carbon (DOC) concentrations, ranging fivefold from approximately 4 to 20 mg C L^−1^ (Sobek, Tranvik, Prairie, Kortelainen, & Cole, 2007; von Wachenfeldt & Tranvik, 2008), so can have very different responses to changes in t‐OM additions because of these differences in overlying water quality (Ask, Karlsson, & Jansson, 2012). We predicted that sediment bacteria would allocate carbon differently, either to enzyme production or CO_2_ production, in a clear and dark lake with increasing additions of terrestrially derived OM, and we used metagenomics to reveal the underlying mechanisms for these changes. We tested our prediction in sediment mesocosms that mirror natural ecosystems in their biogeochemical dynamics and provide a controlled way to replicate t‐OM inputs across lakes with contrasting water quality and clarity (Tanentzap et al., 2017). Microbial community composition and the environment are closely linked, and a major challenge is to decouple these two effects on microbial function (Logue et al., 2016).

## 2 MATERIALS AND METHODS

### 2.1 Study site

Our experiment was deployed in the nearshore region of two lakes near Sudbury, Ontario Canada: Lake Laurentian (46°27′9.74″N, 80°56′35.42″W) and Swan Lake (46°21′58.96″N, 81°3′48.58″W). The sites have minimal human disturbance and are surrounded by similar early‐successional forest, but differ in their overlying water quality. Swan Lake is more oligotrophic than Lake Laurentian, with mean (±*SE*) total phosphorus concentrations (mid‐lake surface grabs taken during our sampling) of 9.3 ± 0.4 µg/L vs. 35.2 ± 2.5 µg/L respectively. Swan also had more than four times higher light levels at the sediment surface. Mean (±*SE*) light levels in Swan were 6,378 ± 149 lx vs. 1,482 ± 28 lx in Laurentian, as measured every 60 min over the duration of our experiment using Hobo UA‐002‐64 light loggers. The loggers were installed on the sediment surface to measure light intensity reaching the sediment–water interface of 24 mesocosms per lake. These differences were consistent with the overlying lake water, as DOC concentrations were 2 mg/L in Swan vs. 7 mg/L in Laurentian. We hereafter refer to the two lakes as the “clear” and “dark” lake, respectively.

### 2.2 Experimental design

We submerged sediment amended with different types of t‐OM on the bottom of the study lakes in the nearshore environment (0.30–0.75 m depth) during July 2015 after Tanentzap et al. (2017). Submergence exposed the experimental sediments to natural overlying water conditions. Briefly, sediments composed of 0%, 5%, 25%, 35%, and 50% t‐OM (dry‐weight basis) and locally sourced inorganic material were mixed with particle sizes and vertical structuring of all material mimicking natural lake sediments (Tanentzap et al., 2017). We then filled 17.5 L, 50.8 × 38.1 × 12.7 (height) cm HDPE containers with 8 cm of sediments (total sediment volume ~15 L). For each t‐OM quantity, material was added in a 1:2, 1:1, or 2:1 dry–mass ratio of deciduous to coniferous litterfall collected from nearby forests. Each of the 5 quantities × 3 qualities combinations was replicated three times in each lake. Mesocosms were arranged in a block design between two sampling bays, submerged in rows at increasing distance from the shoreline, and covered with a 1 × 1 mm nylon mesh screen to standardize shading and resuspension within lakes. Importantly, sediment pore water samples taken from our mesocosms have been found to reflect the biogeochemistry of natural lake sediments (Tanentzap et al., 2017), allowing us to extrapolate our findings to field conditions.

The sediment manipulations interacted with lake conditions to produce experimental gradients in pore water OM quality and quantity within each lake, which we directly measured from optical properties and DOC concentrations, respectively (Supporting Information Figure S1). These pore water dissolved pools were distinct from the overlying lake water and represented the outcomes from mineralization of the added (i.e., sedimented) terrestrial OM. We then followed recommendations to analyze our responses in relation to these continuous gradients rather than the original factorial levels (Cottingham, Lennon, & Brown, 2005). Avoiding the use of categorical variables to represent predictors that are clearly continuous, for example, levels of DOM released from sediment additions, has the added benefit of allowing us to develop predictive models that can demonstrate scientific understanding (see Houlahan, McKinney, Anderson, & McGill, 2017 for further discussion).

### 2.3 Water chemistry and gas sampling

We collected pore water samples from our mesocosms during three sampling periods in June, July and August of 2016. A 3 ml polypropylene syringe was secured horizontally immediately beneath the sediment surface along one side of the HDPE container prior to submergence in the lake. The wall of the syringe that faced the sediment was removed and covered in ca. 250 µm nylon mesh. Each sampling syringe was then connected to 122 cm of nylon tubing that was purged of water before any sample collection.

On each sampling period, we extracted 45 ml of pore water into an airtight 60‐mL syringe. pH was immediately measured with a handheld meter (HI 9126, Hanna Instruments, Woonsocket, RI, USA), before filtering 25 ml of each sample through a 0.5‐µm glass fiber filter (Macherey‐Nagel MN 85/90) and into a 20‐ml glass scintillation vial. Glass vials were preacidified for a sample pH of approximately 2–3 to counter the well‐documented effects of metal quenching of DOM fluorescence that becomes negligible below a pH of 3.0 (Poulin, Ryan, & Aiken, 2014; Spencer, Bolton, & Baker, 2007). In the lab, we measured two widely used DOM metrics using a Cary 60 UV Vis spectrophotometer and a Cary Eclipse fluorescent spectrophotometer (Agilent Technologies, Santa Clara, CA, USA), after analyzing the samples for DOC concentration on a Shimadzu TOC‐5000A (Shimadzu Co, Columbia, MD, USA). The first DOM metric was the specific UV254 absorbance (SUVA), an indication of the average aromatic fraction of DOM per unit DOC. Higher values of SUVA indicate a more reduced state due to intact ring structures that have yet to be oxidized and tend to be HMW DOM (Lavonen et al., 2015). All UV absorbance values were corrected for total iron concentrations, because iron absorbs UV at a similar wavelength to SUVA, and can artificially increase measured SUVA values (O'Donnell, Aiken, Walvoord, & Butler, 2012; Weishaar et al., 2003). Iron was measured using the FerroVer method (Hach Company, 2014) with a Hach DR3900 spectrophotometer (HACH, Loveland, CO, USA). The second metric we measured was the humification index (HIX), for which higher values correspond to longer wavelengths of fluorescing molecules as humification of DOM proceeds (Fellman, Hood, & Spence, 2010). In addition to DOC and DOM, we also measured total dissolved nitrogen (TDN) and total dissolved phosphorus (TDP) using persulfate digestion and ascorbic acid methods, respectively (Hach Company, 2014), with a Hach DR3900 spectrophotometer. TDP and TDN values that were below the detection limit were set to the minimum detection value (0.05 and 1.0 mg/L, respectively).

We also inferred dissolved CO_2_ concentration from the total inorganic carbon concentration, pH and temperature on the day of sample collection from a 45 ml water sample. 2 ml of 0.5 M HCL were injected into the sample before drawing in 15 ml of ambient air, closing the stopcock, and shaking for 2 min to equilibrate the air and acidified sample. 10 ml of the headspace air was then collected in a gas syringe for measurement of CO_2_ concentration on a SRI 8610C gas chromatograph within 48 hr of collection (SRI Instruments, Torrance, CA, USA). Concentration of CO_2_ in the ambient air was measured from a volume of 10 ml and used to correct the headspace measurements. We calculated final pore water concentrations of CO_2_ following methods from Åberg and Wallin (2014) by applying the Bunsen solubility coefficient and ideal gas law, accounting for pH and the ambient air concentration of CO_2_.

### 2.4 Bacterial production

Bacterial production (BP) was measured using ^3^H leucine incorporation after Pace, Giorgio, Fischer, Condon, and Malcom (2004). Duplicate 1.5 ml pore water samples were collected on ice in each of June, July and August 2016 approximately two weeks after sampling water chemistry to analyze how bacteria responded to antecedent DOM conditions. In the laboratory, we added 100 μl of 17.5 nM L‐[4,5–^3^H] leucine (1 mCi/ml, PerkinElmer, Waltham, MA, USA) to each sample. Duplicate “kill” samples were prepared for each combination of OM quality and quantity in each lake. These samples immediately received 0.3 ml of 50% trichloroacetic acid (TCA), killing any live cells, and providing a measure of background ^3^H leucine incorporation that was later subtracted from the incubated values. After incubating for 1 hr, each sample received 0.3 ml of 50% TCA to stop ^3^H leucine uptake. Samples were subsequently centrifuged at 14,243 *g* for 10 min and the supernatant was discarded. Each sample was then washed twice with 5% TCA, dried using a vacuum centrifuge, and stored at −20°C. ^3^H incorporation was later measured using a Beckman Coulter LS6500 liquid scintillation counter (Beckman Coulter, Brea, CA, USA). Prior to counting, each sample received 1 ml of Optiphase Hisafe 2 Scintillation fluid and was vortexed for 30 s. This step was repeated twice more for a total sample volume of 3 ml. Decays per minute measured on the scintillation counter were then converted to BP using standard conversion factors (Bade, Houser, & Scanga, 1998).

### 2.5 Extracellular enzyme activity

We assayed five hydrolytic enzymes (β‐1,4‐glucosidase, β‐^D^‐1,4‐cellobiosidase, β‐1,4‐xylosidase, leucine aminopeptidase, and phosphatase) and two oxidative enzymes (peroxidase and phenol oxidase) in pore water collected in July and August of 2016. Samples were taken one week after water chemistry sampling to test how bacterial enzyme exudation responded to DOM, and one week before BP sampling to test how enzyme activities affected subsequent bacterial C utilization. Pore water samples were assayed directly without dilution (Sinsabaugh et al., 1997), and without buffer to capture the in situ pH (German et al., 2011). Optimal incubation times for hydrolytic enzymes were determined by standard time trials as recommended and described in German et al. (2011). We found these times to be 2 hr for β‐1,4‐glucosidase and leucine aminopeptidase and 1 hr for β‐^D^‐1,4‐cellobiosidase, β‐1,4‐xylosidase, and phosphatase. Timings of oxidative incubations were performed as suggested in Sinsabaugh et al. (2003). Fluorescence and absorbance were read on a Synergy H1 microplate reader (BioTek, Winooski, VT, USA). β‐^D^‐1,4‐cellobiosidase, β‐1,4‐xylosidase, and β‐1,4‐glucosidase were summed as total hydrolase activity, as these enzymes collectively target structural components of plant‐derived OM (Emilson, Kreutzweiser, Gunn, & Mykytczuk, 2016). Similarly, peroxidase and phenol oxidase activity were expressed as total oxidase activity because they have a similar functional role (Sinsabaugh, 2010; Sinsabaugh et al., 2003).

### 2.6 Metagenomics shotgun sequencing

DNA was extracted in duplicate from each mesocosm in August 2016 using a PowerSoil DNA Isolation Kit (MoBio Laboratories Inc, Carlsbad, CA, USA), and following the manufacturer's protocol. Sequencing libraries were prepared with 1 ng of genomic DNA per sample using the Nextera XT DNA Sample Preparation and dual‐barcoding with Nextera XT Indexes (Illumina, San Diego, CA, USA) following the manufacturer's instructions. Libraries were quantified on a Qubit 3.0 Fluorometer (ThermoFisher Scientific, Waltham, MA, USA) and on a Bioanalyzer HS DNA chip (Agilent, Santa Clara, CA) and pooled in equimolar concentrations into a single sample. Samples were sequenced on an Illumina platform using a NextSeq 500/550 Mid Output Kit v2 (300 cycles, paired‐end).

Raw sequences were processed at a read depth of approximately 3.3 million reads per sample following the European Molecular Biology Laboratory‐European Bioinformatics Institute (EBI) pipeline version 3.0 (Mitchell et al., 2016). In brief, the SeqPrep tool (https://github.com/jstjohn/SeqPrep, version 1.1) was used to merge paired‐end overlapping reads, Trimmomatic (Bolger, Lohse, & Usadel, 2014, version 0.35) was used to trim low quality ends and sequences with >10% undetermined nucleotides, and sequences <100 nucleotides were removed using Biopython (Cock et al., 2009, version 1.65). Sequences were functionally annotated by predicting coding sequences (pCDS) above 60 nucleotides with FragGeneScan (Rho, Tang, & Ye, 2010, version 1.20). Read matches were then generated against pCDS using a subset of databases from InterProScan (Jones et al., 2014, version 5.19‐58.0) and summarized using the Gene Ontology terms. Sequences were taxonomically annotated with QIIME (Caporaso et al., 2010, version 1.9.1). Representative 16S sequences were classified using the SILVA reference database (Quast et al., 2013, release 128) at 97% sequence identity following the open‐reference Operational Taxonomic Unit (OTU) picking method with reverse strand matching enabled (Caporaso et al., 2010). The sequences were deposited in EBI Metagenomics under the project accession number ERP019980. We normalized both the functional and OTU datasets by transforming the abundance of annotated sequences to abundances relative to the per sample total to account for sequencing bias (McMurdie & Holmes, 2014; Vincent, Derome, Boyle, Culley, & Charette, 2017). We focused our analysis on the subset of functional genes that matched the groupings of hydrolytic (“cellulase activity,” “glucosidase activity,” and “xylan 1,4‐beta‐xylosidase activity”) and oxidative enzymes (“peroxidase activity” and “catechol 1–2‐dioxygenase activity”) measured for extracellular activity, as well as genes required to catabolize aromatic DOM (“aromatic compound catabolic process”) and genes for oxidoreductase activity involving oxygen (“oxidoreductase activity, acting on paired donors, with incorporation or reduction of molecular oxygen”), an important step in the decomposition of aromatics (Sinsabaugh, 2010).

### 2.7 Statistical analysis

We tested the effects of DOM quantity and quality on bacterial responses using the linear mixed‐effects models with the *lmer* function in the *lme4* package in R v3.2 (R Core Team, 2017). Bacterial responses included BP, CO_2_ concentrations, hydrolase activity, oxidase activity, phosphatase activity, and leucine amino peptidase activity. We transformed responses with a quarter‐power rather than logarithmically because small values in some of the responses resulted in heavy left‐tail‐distributions with the latter. Each response was then predicted as a function of DOC, SUVA and HIX, in addition to sampling month and environmental variables pH, TDN and TDP. The model for CO_2_ also included BP as a predictor because CO_2_ is a combined product of bacterial respiration and photo‐mineralization, which can account for up to 50% and 90% of total production respectively, depending on environmental conditions (Kirchman, 2013; Ward & Cory, 2016). We accounted for the blocking design of our experiment, whereby the mesocosms were distributed around two sampling bays, by including bay as a fixed factor. We also accounted for repeated measurements of the same mesocosm and blocking row to account for proximity to shoreline and decreasing depth by including these factors as random effects. Finally, we allowed mean responses to vary between the dark and light lake (i.e., lake fixed effect) and tested whether the effects of DOC, DOM quality, and BP (for CO_2_ response) differed between lakes with contrasting light penetration (i.e., a statistical interaction). Temperature was excluded from the models because it did not vary among mesocosms within lakes (mean ± *SE* of 15.85 ± 0.03C and 14.0 ± 0.02C in the dark and clear lake, respectively).

We determined the best‐fitting model that explained our responses using backwards stepwise selection. Models were compared using the Akaike information criterion (AIC) and we sequentially removed predictors that did not increase AIC by more than 2, ensuring the more parsimonious models also never had AICs that were more than 2 values higher than the lowest observed value across the entire model set. Main effects were only dropped after their interactions. We assessed the significance of factors in the best supported model with *p*‐values calculated from a *t*‐distribution using the R package *lmerTest* (Kuznetsova, Brockhoff, & Bojesen, 2016). Degrees of freedom were adjusted with the Kenward–Roger method, which reduces bias in small sample settings (Kenward & Roger, 1997).

We also analyzed the relative abundances of OTUs and functional genes relating to t‐OM degradation to identify potential reasons for lake‐dependent bacterial responses. First, we tested for a compositional difference between lake bacterial communities using a permutational multivariate analysis of variance (perMANOVA) with Bray–Curtis distances between samples. We also tested whether compositional differences were associated with sediment pore water DOC, SUVA, HIX, and pH within mesocosms of each lake by including these variables in the perMANOVA. Significance of marginal effects was assessed with 1,000 permutations of the raw data, which were restricted to each lake for the covariates, using the *adonis2* function in the R package *vegan* version 2.5‐2 (Oksanen et al., 2018). We did not include TDN and TDP in these analyses to minimize collinearity; this issue was not a concern for linear mixed‐effects models as none of the parameter estimates were strongly intercorrelated (*r* > 0.75). Differences in OTU relative abundance between lakes were visualized with a nonmetric multidimensional scaling (NMDS) ordination. We also used a perMANOVA with Bray–Curtis distances to test whether the relative abundances of the subset of functional genes could be explained by environmental variables and lake identity. Again, we visualized statistically significant factors (*p < *0.05) using a NMDS ordination. In a separate perMANOVA, we tested whether the original manipulations of t‐OM quantity and quality were also correlated with the composition of pore water bacterial communities and functional genes. Finally, we tested if the relative abundance of the subset of functional genes differed on average between lakes using paired *t* tests. We paired mesocosms that were in the same position in our experimental block design but located in different lakes.

Finally, we quantified the difference in bacterial community composition between the natural sediments of the two study lakes at the start of the experiment. We calculated the Bray–Curtis dissimilarity index between six pooled samples from each lake, where a value of 0 indicated communities were identical and a value closer to 1 indicated communities were entirely different, that is, no overlapping OTUs (Bloom, 1981).

## 3 RESULTS

### 3.1 Bacterial responses to t‐OM addition depended on lake clarity

**Figure 1:**
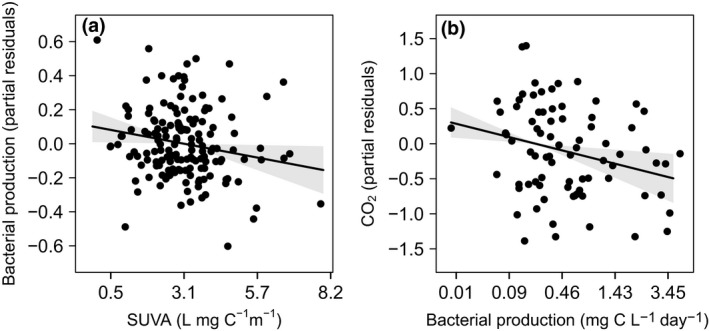
Energetic tradeoffs in the dark but not the clear lake. (a) BP decreased with specific ultraviolet absorbance (SUVA) across both lakes, resulting in (b) more CO2 production where BP was lower, but only in the dark lake. Solid lines are mean effects ±95% confidence intervals

**Figure 2:**
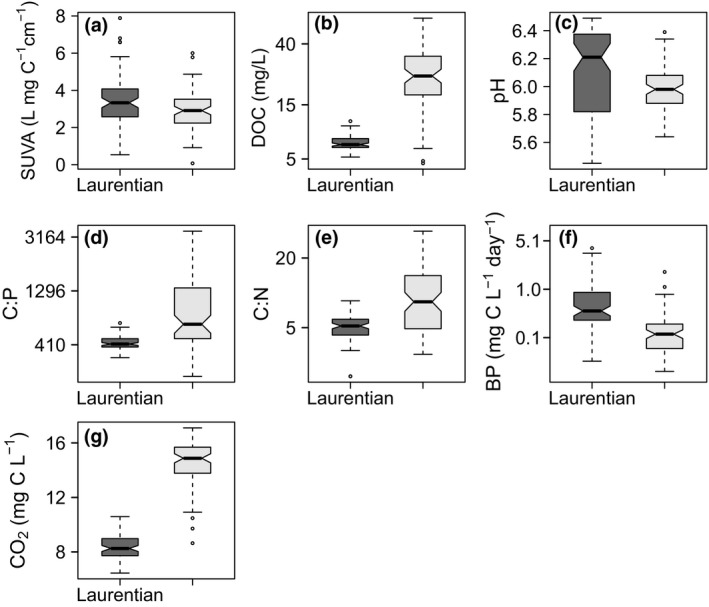
Pore water differed between mesocosms in the dark (LAU) and clear (SWA) lake. The dark lake had (a) more SUVA, (b) lower DOC concentrations, (c) slightly higher pH, (d) lower C:P and (e) C:N ratios, (f) more BP, and (g) less CO_2_ production. Nonoverlapping notches indicate differences in the two medians based on 95% confidence intervals (Chambers, Cleveland, Kleiner, & Tukey, 1983). The upper and lower whiskers extended 1.5 times the interquartile range, with points outside of this range plotted

We found that BP decreased with the aromatic fraction of DOM (i.e., SUVA) across both lakes (*t* = −2.04, *df* = 156, *p* = 0.042), such as if the bacteria expended more energy to break down aromatic DOM than to grow. For example, a 25% increase above the mean aromaticity of 3.37 L mg‐C^−1^ cm^−1^ decreased BP below its mean of 0.54 g C L^−1^ day^−1^ by 14.5% (Figure 1a). The decrease in BP with an increasing fraction of aromatic pore water DOM in the dark lake was associated with a small increase in CO_2_ production (*t* = −2.68, *df* = 158, *p = *0.008, Figure 1b), suggesting that bacteria in the dark lake were incurring a higher metabolic and respiratory cost associated breaking down aromatic OM. Specifically, CO_2_ increased by 2% above the mean value of 8.59 mg‐CO_2_ L^−1^ with a 25% decrease in BP. In contrast to the dark lake, greater UV exposure at the sediment–water interface in the clear lake may have led to photo‐oxidation of DOC that increased CO_2_ production (lake effect: *t* = 13.38, *df* = 155, *p* < 0.001). Greater photo‐degradation in the clear lake may have also resulted in a lower concentration of aromatic DOM relative to the dark lake (two‐sample *t* test: *t* = −2.64, *df* = 143, *p* = 0.009, Figure 2a). Despite the higher pore water DOC concentration in the clear lake, of which less was aromatic and presumably more was LMW C (Figure 2a,b), BP was lower on average (*t* = −6.54, *df* = 120, *p* < 0.001). The lower BP likely arose because of nutrient limitation that was inseparable from the lake‐level effect (Figure 2d‐e). On average, the clear lake always had 13.6% and 15.0% less available N and P, respectively, relative to C (i.e., higher pore water C:N and C:P ratios) when comparing the same mesocosms between lakes (*t* = 7.59, *df* = 118, *p < *0.001; *t* = 16.74, *df* = 100, *p < *0.001). Full results of model selection are given in Supporting Information Table S1.

### 3.2 Extracellular enzyme production enabled aromatic OM decomposition in the dark lake

**Figure 3:**
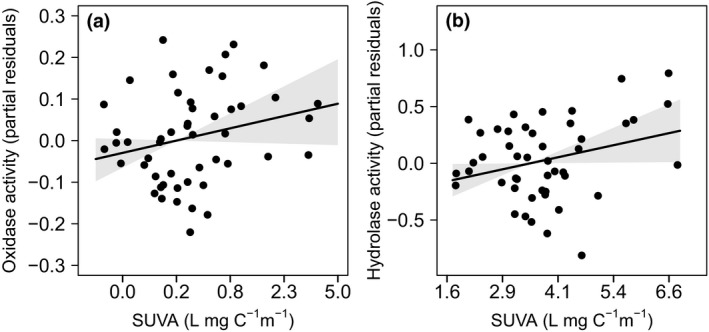
Enzyme activities increased with aromaticity of DOM in the dark lake. (a) Oxidase and (b) hydrolase activity increased with specific UV254 absorbance (SUVA). Solid lines are mean effects ±95% confidence intervals

The decrease in bacterial productivity in the dark lake corresponded with a positive association between hydrolytic and oxidative enzyme activity and the fraction of aromatic pore water DOM. Bacteria were presumably limited for LMW substrates in the dark lake and subsequently produced more hydrolytic and oxidative enzymes with higher fractions of SUVA (*t* = 2.09, *df* = 99, *p* = 0.039 and *t* = 4.27, *df* = 111, *p < *0.001, respectively; Figure 3). For example, a 25% increase in the mean aromatic fraction of 3.37 L mg‐C^−1^ cm^−1^ increased oxidative and hydrolytic enzyme activity by 14.6% and 11.4% above the mean values of 0.05 µmol ml^−1^ hr^−1^ and 230.0 ηmol ml^−1^ hr^−1^, respectively. There was no response to aromaticity in the clear lake for oxidative (*t* = −1.33, *df* = 112, *p* = 0.100) or hydrolytic enzymes (*t* = 0.66, *df* = 106, *p = *0.419), suggesting that bacteria were utilizing less terrestrially derived OM for growth. Both phosphatase and leucine aminopeptidase had no response to DOM quality or DOC concentration (Supporting Information Table S2).

### 3.3 Different bacterial communities underlie varying responses to t‐OM addition

**Figure 4:**
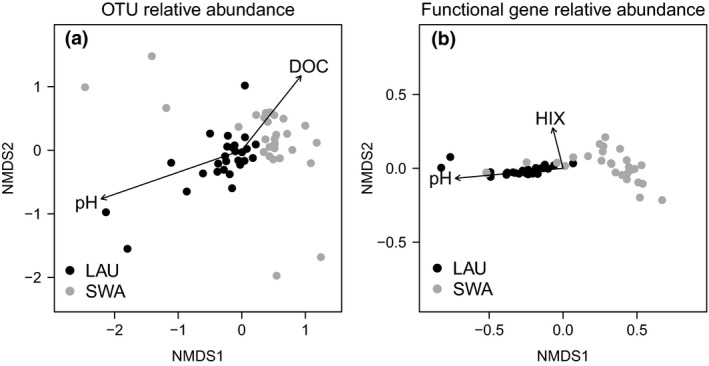
Bacterial community and functional composition differ between the dark (LAU) and clear (SWA) lake. (a) Nonmetric multidimensional scaling (NMDS) of OTU abundance in individual mesocosms (stress = 0.13), where lake, pH, and dissolved organic carbon (DOC) were correlated with OTU relative abundances within mesocosms (for all, *p* < 0.05 with perMANOVA). (b) NMDS of relative abundance of functional genes related to t‐OM degradation (stress = 0.02), where lake, pH, and HIX and were correlated with functional gene composition within mesocosms (for all, *p* < 0.05 with perMANOVA)

**Figure 5:**
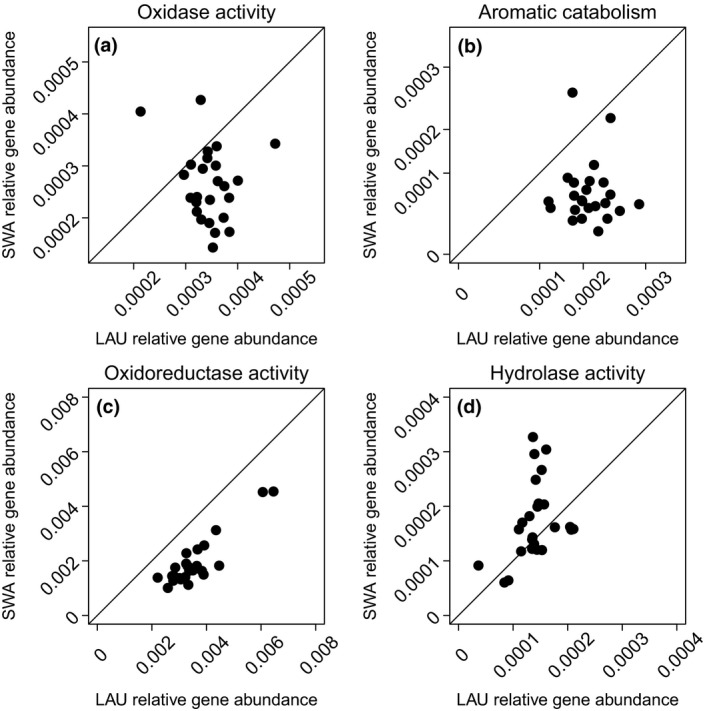
Functional genes involved in OM degradation were relatively more abundant in the dark than clear lake. Genes considered were those involved in (a) oxidase activity, which includes the sum of peroxidase and catechol, 1‐2‐dioxygenase gene abundances, (b) aromatic catabolism, (c) oxidoreductase activity, and (d) hydrolase activity, which is the sum of glucosidase, cellobiosidase, and xylosidase gene abundances. The line indicates a 1:1 ratio. Points above or below the line indicate a higher abundance of genes in the clear and dark lake, respectively

We found evidence that, in addition to differences in available nutrients, bacterial activity might have also differed between the two lakes because of underlying differences in community composition and subsequent functional potential to decompose t‐OM. The relative abundance of pore water bacterial OTUs differed significantly between the two lakes across all mesocosms (*F*_1,49_ = 2.01, *p = *0.001, Figure 4a). Correspondingly, we found that genes relating to oxidative enzymes (*t* = 4.42, *df* = 24, *p < *0.001), aromatic catabolism (*t* = 9.22, *df* = 24, *p* < 0.001) and oxidoreductase activity (*t* = 18.71, *df* = 24, *p* < 0.001) were on average 1.1–3.0 more times abundant in the darker lake where microbial activity responded more strongly to increasing fractions of aromatic OM (Figure 5a–c). There were slightly higher abundances of hydrolytic genes in the clear lake (*t* = −2.38, *df* = 24, *p* = 0.025, Figure 5d), but because enzyme expression and exudation may be more strongly induced by low concentrations of LMW substrates than the presence of HMW DOM, the clear lake bacterial communities did not produce more enzymes in response to increasing DOC quantity (Supporting Information Table S2).

The observed differences in bacterial community composition and function may have arisen because in‐lake processes, including photo‐oxidation at the sediment–water interface, caused the added t‐OM to diverge between lakes. Communities varied with pore water DOC (*F*_1,49_ = 1.42, *p = *0.037) and pH gradients in each lake (*F*_1,49_ = 1.48, *p = *0.009; Figure 4a), which themselves differed between lakes (Figure 2). Functional genes capable of degrading inputs of t‐OM also differed between the two lakes (*F*_1,49_ = 4.13, *p* = 0.035) and varied along gradients of humic DOM (*F*_1,49_ = 4.63, *p* = 0.030) and pH in sediment pore water of each lake (*F*_1,49_ = 5.24, *p* = 0.031; Figure 4b). Consistent with these results, we found that the composition of pore water bacterial communities changed with the original quantity and quality of t‐OM added in each lake (*F*_1,50_ = 2.75, *p* = 0.016; *F*_3,50_ = 3.48, *p* = 0.001, respectively). The presence of functional genes mirrored these results *(F*_1,50_ = 2.22, *p* = 0.001; *F*_3,50_ = 1.80, *p* = 0.001, respectively). Both lakes also had very different resident bacterial communities at the start of our experiment, as expected if the existing environment was responsible for altering the quality of t‐OM received by the sediment bacteria (Bray–Curtis dissimilarity between lakes = 0.90).

## 4 DISCUSSION

Our analysis of sediment pore water at the interface between sediments and the overlying water column in a dark and a clear lake shows that environmental conditions interact with community composition to control the functioning of bacterial communities and ultimately their responses to t‐OM additions. Despite the same t‐OM being added to the experimental sediments in each lake, biogeochemical processing differed. More DOC and CO_2_ and less aromatic DOM was produced in the sediment pore water of the clear lake than in the dark lake. The bacterial community in the dark lake had higher relative abundances of functional genes related to t‐OM decomposition, enabling greater enzyme activity. A relatively lower abundance of these functional genes limited the bacterial response to aromatic DOM in the clear lake. The overall higher amount of CO_2_ production in the clear lake suggested that the bacterial community instead mineralized the photo‐oxidized, LMW DOM (Hessen, 1992; Moran & Zepp, 1997). Mineralization likely arose because of nutrient limitation relative to the dark lake, where terrestrial nutrient inputs were higher and bacterial production could be sustained at a higher overall level (Figure 2f; López‐Urrutia & Morán, 2007; Reche et al., 1998). As future influxes of t‐OM increase across boreal lakes (Creed et al., 2018; Sobek et al., 2009), these water clarity‐dependent responses suggest CO_2_ release may be greater in clear lakes with high levels of photo‐oxidation at the sediment surface (Lapierre, Guillemette, Breggren, & del Giorgio, 2013). Dark lakes may instead experience a decrease in primary production and shift toward retaining rather than mineralizing terrestrial carbon, such as by burying it in sediment (Gudasz et al., 2017; Isidorova et al., 2015; Seekell et al., 2015). However, over the longer term, the darkening of clear lakes will reduce light exposure on the sediment surface, increasing OM burial and encouraging bacterial communities to develop in sediment pore water that can utilize this material (Judd, Crump, & Kling, 2007; Rofner et al., 2017).

The ability for bacteria in the dark lake sediments to break down increasing fractions of terrestrially derived organic matter was possible because they had the genes to produce hydrolyzing and oxidizing enzymes (Judd et al., 2007; Ward & Cory, 2016). Although this may have allowed the dark lake bacteria to access nutrients and bioavailable growth‐promoting amino acids associated with t‐OM, the metabolic cost of producing enzymes likely resulted in less growth (Logue et al., 2016; López‐Urrutia & Morán, 2007; Roiha, Peura, Matheiu, & Rautio, 2016; Yamashita, Fichot, Shen, Jaffé, & Benner, 2015). Additionally, bacteria in the dark lake had a limited availability of LMW substrates from photosynthetically derived and photo‐oxidized OM (Bertilsson & Tranvik, 1998; Karlsson et al., 2009). The availability of LMW substrates was further limited by the rate at which extracellular enzymes could oxidize and hydrolyze HMW t‐OM. As there was no resulting pool of excess LMW substrates to facilitate metabolic cycling (Chróst, 1991; Hessen, 1992), the sediment bacterial community in the dark lake consequently did not produce excess CO_2_ in addition to the respiration required for basal metabolic function, and instead produced extracellular enzymes to degrade DOM with a greater terrestrial signature (Chróst & Rai, 1993; Russell, 2007; Sinsabaugh, 2010).

In the clear lake, bacterial metabolic cycling of a LMW DOC pool and direct photo‐mineralization at the sediment surface likely accounted for higher CO_2_ production. Previous work has shown that sunlight exposure photo‐degrades aromatic DOM and leads to higher concentrations of LMW compounds (Anesio, Graneli, Aiken, Kieber, & Mopper, 2007; Bertilsson & Tranvik, 1998; Sulzberger & Durisch‐Kaiser, 2009). This process could lead to more bioavailable carbon and elevated CO_2_ production (Lapierre et al., 2013; Lapierre & del Giorgio, 2014). However, growth of sediment bacteria in the clear lake may have been limited by the lower availability of N and P relative to C (per mass of DOC) (Figure 2d,e; Goldman et al., 1987; Reche et al., 1998; Smith & Prairie, 2004; Sterner, Elser, Fee, Guildford, & Chrzanowski, 1997; Sterner et al., 1998), despite the available LMW compounds. This nutrient limitation can explain why communities would have metabolically cycled C instead of allocating it to biomass, thus producing more CO_2_ than the dark lake (del Giorgio & Cole, 1998; Hessen, 1992; Karlsson, Jansson, & Jonsson, 2002; Reche et al., 1998; Smith & Prairie, 2004; Zwart et al., 2016). Therefore, increasing t‐OM influx may have varied effects on in‐lake carbon cycling (Lapierre & del Giorgio, 2014). Photo‐mineralization can also produce up to 90% of CO_2_ in aquatic systems, and may have accounted for some of the CO_2_ observed in the clear lake pore water (Ward & Cory, 2016). This high rate of photo‐oxidation in the clear lake as opposed to the dark lake also altered the mesocosm pore water DOM quality, which may have shifted the composition of the clear lake bacterial community to taxa that were better able to degrade photoproducts, whereas the dark lake community may have been predisposed to degrade t‐OM (Judd et al., 2007; Lønborg et al., 2016; Sharpless et al., 2014; Ward & Cory, 2016).

Our results add to the growing evidence that terrestrially derived OM is not recalcitrant (Judd et al., 2006; Kellerman, Dittmar, Kothawala, & Tranvik, 2014; Kritzberg, Langenheder, & Lindstrom, 2006; McCallister & del Giorgio, 2012; Roiha et al., 2016). Rather, t‐OM can undergo photo‐induced transformations and be degraded by EEAs, leading to CO_2_ production in lake sediments (Karlsson et al., 2012). Although bacterial activity was not directly stimulated by aromatic OM, it was relatively higher in lake sediments dominated by aromatic OM, thereby suggesting that the terrestrially sourced fraction of DOM is relatively bioavailable and has a potentially high turn‐over rate (Karlsson et al., 2009; Kleber et al., 2011). These findings challenge the paradigm that t‐OM is highly degraded, recalcitrant, and otherwise unavailable to microbial degradation due to its chemical composition (Judd et al., 2007; McCallister & del Giorgio, 2012; Sollins, Homann, & Caldwell, 1996; Zwart et al., 2016). Consequently, we suggest that t‐OM inputs to lakes will not remain unchanged in lake sediments, but will be altered by microorganisms and photo‐processes, which may change carbon budgets and/or transfer heterotrophic productivity to higher trophic levels (Cole, Caraco, Kling, & Kratz, 1994; Forsström, Roiha, & Rautio, 2013; McCallister & del Giorgio, 2012; Tanentzap et al., 2014). More broadly, our results advance previous work by showing how the fate of t‐OM in lake sediments will ultimately depend on local environmental conditions and the bacterial community's genetic potential to degrade different OM qualities.

We found that variation in the functional genes of sediment bacteria and nutrient availability could explain differences in t‐OM utilization between two lakes of contrasting water clarity. This finding suggests that future inputs of t‐OM, such as those observed across the Boreal region (Monteith et al., 2007; Pregitzer, Zak, Burton, Ashby, & Macdonald, 2004; Solomon et al., 2015), can have dramatically different outcomes for whole‐lake C cycles depending on lake‐specific characteristics (Ruiz‐Gonzalez et al., 2015; Traving et al., 2016; Zwart et al., 2016). In clear lakes, increased inputs of t‐OM may induce a positive feedback loop, whereby increased CO_2_ production stimulates primary production, leading to additional inputs of LMW compounds from algae (Hare et al., 2007; Schippers, Lürling, & Scheffer, 2004). In contrast, elevated inputs of t‐OM in dark lakes could lead to an increase in organic matter burial and an increasingly heterotrophic food web (Forsström et al., 2013; Gudasz et al., 2017). Future predictions of how t‐OM will impact whole‐lake processes clearly need to consider lake‐specific characteristics, such as water clarity and nutrient availability.
