# Supplementary material for: Feasting on terrestrial organic matter: Dining in a dark lake changes microbial decomposition
Source: Glob Chang Biol. 2018 Aug 26;24(11):5110–22. doi: 10.1111/gcb.14391 (PMC6220883; doi:10.1111/gcb.14391)
Supplement: Supplementary file 1 [file GCB-24-5110-s001.docx]

**Supplementary Information**

**Table S1.** Model selection results. Predictors were sequentially removed so that the AIC for the most parsimonious model was not more than 2 units greater than the lowest AIC value observed across the candidate set. Interactions between lake and continuous predictors are indicated by a colon (:). All models included mesocosm id and blocking row as random effects, and blocking bay and sampling month as fixed effects. Minus signs denote variables that were progressively removed from the full model. Where only the fully saturated model is reported, any dropped parameter increased the AIC value by >2.

| **Model** | **Number of parameters** | **ΔΑΙC** |
| --- | --- | --- |
| BP predictors: SUVA + HIX + DOC + pH + TDP + TDN + HIX:lake + DOC:lake + SUVA:lake | | |
| Full model | 14 |  |
| -SUVA:lake | 13 | -2 |
| -TDP | 12 | -1.8 |
| -TDN | 11 | 1.8 |
| Final BP predictors: DOC + pH + SUVA + HIX + HIX:lake + DOC:lake | | |
| **Model** | **Number of parameters** | **ΔΑΙC** |
| Oxidase predictors: SUVA + HIX + DOC + TDN + TDP + pH + HIX:lake + SUVA:lake + DOC:lake | | |
| Full model | 14 |  |
| -HIX:lake | 13 | -1.8 |
| -DOC:lake | 12 | -1.4 |
| -DOC | 11 | -1.7 |
| -TDN | 10 | 0.2 |
| Final oxidase predictors: TDP + pH + SUVA+ HIX + SUVA:lake | | |
| **Model** | **Number of parameters** | **ΔΑΙC** |
| Hydrolase predictors: SUVA + HIX + DOC + TDN + TDP + pH + HIX:lake + DOC:lake + SUVA:lake | | |
| Full model | 14 |  |
| -DOC:lake | 13 | -1.2 |
| -HIX:lake | 12 | -0.7 |
| -HIX | 11 | 0.7 |
| -TDP | 10 | 0.5 |
| -DOC | 9 | -0.8 |
| Final hydrolase predictors: SUVA + TDN + pH + SUVA:lake | | |
| **Model** | **Number of parameters** | **ΔΑΙΧ** |
| Phosphatase predictors: SUVA + HIX + DOC + TDN + TDP + pH + HIX:lake + DOC:lake + SUVA:lake | | |
| Full model | 14 |  |
| -TDP | 13 | -2.3 |
| -TDN | 12 | -1.7 |
| -pH | 11 | -2.0 |
| -DOC:lake | 10 | -1.8 |
| -SUVA:lake | 9 | -1.4 |
| -HIX:lake | 8 | -0.9 |
| -SUVA | 7 | -1.8 |
| -HIX | 6 | +0.1 |
| Final phosphate predictors: DOC | |  |
| **Model** | **Number of parameters** | **ΔΑΙC** |
| Leucine predictors: SUVA + HIX + DOC + TDN + pH + SUVA:lake + HIX:lake + DOC:lake | | |
| Full model | 14 |  |
| -TDP | 13 | -1.1 |
| -TDN | 12 | -0.6 |
| -pH | 11 | -1.2 |
| -DOC:lake | 10 | -1.3 |
| -SUVA:lake | 9 | -0.9 |
| -DOC | 8 | -2.0 |
| -SUVA | 7 | -2.0 |
| Final leucine predictors: HIX + HIX:lake | |  |
| **Model** | **Number of parameters** | **ΔΑΙC** |
| CO2 predictors: BP+ SUVA + HIX + DOC + TDN + TDP + pH + HIX:lake + DOC:lake + SUVA:lake + BP:lake | | |
| -TDP | 15 | -1.9 |
| -TDN | 14 | -1.8 |
| -HIX:lake | 13 | -1 |
| -HIX | 12 | -1.7 |
| -pH | 11 | -2 |
| -SUVA:lake | 10 | -0.1 |
| -SUVA | 9 | -0.1 |
| Final CO2 predictors: BP+ DOC + BP:lake + DOC:lake | | |

**Table S2.** Effect sizes, *t* values, degrees of freedom (df) and *p* values for best-supported models. Interactions between continuous predictors and lake are indicated by a colon (:). Predictors that differ in response by lake are indicated by a colon (:) and either “Dark” or “Clear” to specify the absolute response. Degrees of freedom vary for predictors within a model due to a correction for unequal variances. Bolded *p* values for predictors indicate significant effects.

| **Response** | **Predictor** | **Mean Effect ± [SE]** | ***t*** | **df** | ***p* value** |
| --- | --- | --- | --- | --- | --- |
| Bacterial production | Lake | -0.07 ± 0.12 | -0.53 | 170 | 0.596 |
|  | SUVA | -0.04 ± 0.02 | -2.04 | 156 | **0.042** |
|  | pH | 0.09 ± 0.02 | 3.68 | 159 | **<0.001** |
|  | DOC:Dark | -0.29 ± 0.14 | -1.96 | 170 | 0.052 |
|  | DOC:Clear | 0.01 ± 0.03 | 0.375 | 124 | 0.98 |
|  | HIX:Dark | -0.13 ± 0.05 | -2.39 | 168 | **0.018** |
|  | HIX:Clear | -0.04 ± 0.02 | -2.15 | 148 | **0.033** |
| CO2 production | Lake | 2.26 ± 0.39 | 7.98 | 151 | **<0.001** |
|  | DOC:Dark | 3.29 ± 1.06 | 3.01 | 149 | **0.003** |
|  | DOC:Clear | 1.45 ± 0.10 | 13.38 | 155 | **<0.001** |
|  | BP:Dark | -0.30 ± 0.11 | -2.68 | 158 | **0.008** |
|  | BP:Clear | -0.04 ± 0.12 | -0.30 | 152 | 0.507 |
| Hydrolase activity | Lake | -0.17 ± 0.07 | -1.11 | 104 | 0.270 |
|  | pH | -0.21 ± 0.07 | -3.37 | 104 | **0.001** |
|  | TDN | 0.24 ± 0.07 | 3.13 | 104 | **0.002** |
|  | SUVA:Dark | 0.16 ± 0.07 | 2.09 | 99 | **0.039** |
|  | SUVA:Clear | 0.05 ± 0.08 | 0.66 | 106 | 0.419 |
| Oxidase activity | Lake | 0.37 ± 0.039 | 8.85 | 108 | **<0.001** |
|  | TDP | 0.04 ± 0.02 | 2.32 | 111 | **0.022** |
|  | HIX | 0.06 ± 0.02 | 3.19 | 111 | **0.002** |
|  | pH | -0.09 ± 0.02 | -4.39 | 103 | **<0.001** |
|  | SUVA:Dark | 0.10 ± 0.02 | 4.27 | 111 | **<0.001** |
|  | SUVA:Clear | -0.04 ± 0.02 | -1.33 | 112 | 0.100 |
| Phosphatase activity | Lake | -0.59 ± 0.12 | -4.48 | 74 | **<0.001** |
|  | DOC | -0.12 ± 0.06 | -1.95 | 72 | 0.055 |
| LEU aminopeptidase activity | Lake | -0.05 ± 0.11 | -0.48 | 88 | 0.633 |
|  | HIX:Dark | -0.25 ± 0.15 | -1.57 | 92 | 0.120 |
|  | HIX:Clear | 0.08 ± 0.06 | 1.18 | 101 | 0.242 |

**Figure S1**. Gradients in pore water biogeochemistry unexpectedly arose one-year after additions of different terrestrial organic matter (OM) quantity and quality to experimental sediments. Points are mean ± standard error across 3 monthly measurements taken during 2016 for 3 experimental replicates at each of 3 OM quantity (% dry-weight basis, dwt) and 3 OM quality treatments (expressed as ratios of coniferous, con, to broadleaf, bro, forest litter) in the dark (black points) and clear (white points) lakes for: **(a)** bacterial production (BP), **(b)** dissolved organic carbon (DOC), **(c)** specific ultraviolet absorbance (SUVA), **(d)** CO_2_ production, **(e)** C:N ratio, and **(f)** C:P ratio. We also sampled 3 control replicates of no OM addition.
